# Brain-Inspired Coding of Robot Body Schema Through Visuo-Motor Integration of Touched Events

**DOI:** 10.3389/fnbot.2019.00005

**Published:** 2019-03-07

**Authors:** Ganna Pugach, Alexandre Pitti, Olga Tolochko, Philippe Gaussier

**Affiliations:** ^1^ETIS Laboratory, University Paris-Seine, CNRS UMR 8051, University of Cergy-Pontoise, ENSEA, Cergy-Pontoise, France; ^2^Faculty of Electric Power Engineering and Automation, National Technical University of Ukraine Kyiv Polytechnic Institute, Kyiv, Ukraine

**Keywords:** body schema, multimodal integration, artificial skin, parietal cortex, gain-field neurons, peri-personal space, visual reaching, non-linear mixed-selectivity

## Abstract

Representing objects in space is difficult because sensorimotor events are anchored in different reference frames, which can be either eye-, arm-, or target-centered. In the brain, Gain-Field (GF) neurons in the parietal cortex are involved in computing the necessary spatial transformations for aligning the tactile, visual and proprioceptive signals. In reaching tasks, these GF neurons exploit a mechanism based on multiplicative interaction for binding simultaneously touched events from the hand with visual and proprioception information.By doing so, they can infer new reference frames to represent dynamically the location of the body parts in the visual space (i.e., the body schema) and nearby targets (i.e., its peripersonal space). In this line, we propose a neural model based on GF neurons for integrating tactile events with arm postures and visual locations for constructing hand- and target-centered receptive fields in the visual space. In robotic experiments using an artificial skin, we show how our neural architecture reproduces the behaviors of parietal neurons (1) for encoding dynamically the body schema of our robotic arm without any visual tags on it and (2) for estimating the relative orientation and distance of targets to it. We demonstrate how tactile information facilitates the integration of visual and proprioceptive signals in order to construct the body space.

## 1. Introduction

The body schema is the perception that each individual has of his own body in space. The acquisition of this body schema during infancy helps to learn a structural organization of the body parts and their visual shape, to establish the boundaries of the body and to situate better its physical limits (Gliga and Dehaene-Lambertz, 2005; Klaes et al., 2015; Marshall and Meltzoff, 2015; Bhatt et al., 2016; Jubran et al., 2018). Gradually, the body schema grows to enhance spatial awareness to objects (reaching and grasping) (Van der Meer, 1997; Corbetta et al., 2000) and to others (self-other differentiation, eye-gaze; Deák et al., 2014). In order to guide the movement of the body in space and to allow interaction with an immediate environment, the brain must constantly monitor the location of each body part at different postures and to analyze the spatial relationship between body parts and neighboring objects.This process requires the integration of proprioceptive, tactile, visual, and even auditory information to align the different reference frames from each other; for instance, eye-, hand-, torso-, or head-centered reference frames. Although many data are collected from neurosciences, the mechanisms behind multimodal integration from raw input for aligning the different reference frames and for constructing this body schema are still under investigation and several models and mechanisms have been proposed; c.f., (Taira et al., 1990; Burnod et al., 1992; Sakata et al., 1995; Caminiti et al., 1998; Avillac et al., 2005; Borra et al., 2017). For robotics, endowing to robots a body schema could help in reaching and grasping tasks or in developing a sense of spatial awareness in order to interact physically and socially with persons.

Many neuroscience studies have focused on how various sensory modalities can be combined and integrated to achieve the perception of limb location and the representation of space immediately around the body (i.e., the peripersonal space). Graziano and Botvinick (2002) presented in one study two visions of how the brain represents the body through neurophysiology and psychology.The psychological approach emphasizes the multisensory nature of body representation and has shown that touch and proprioception are combined in a sophisticated mental schema from the body. In contrast, neurophysiology focuses on proprioception, a component of the representation of the body, and focuses primarily on the use of proprioception in the movement control.

In a dynamic environment, the characterization of the peripersonal space of a complex animal is fundamental for reacting appropriately when an object enters in it. The natural reaction could be either grasping or approaching the object if it is of interest, or avoiding it if it represents a danger (Graziano and Aflalo, 2007). Therefore, the brain integrates different information from visual, auditory or somatosensory systems to ensure an effective representation of the body and peripersonal space (Holmes and Spence, 2004).

The peripersonal space is defined as the space that immediately surrounds our body (Rizzolatti et al., 1997). The neuronal representation of the peripersonal space is constructed through a network of cortical and subcortical brain zones. To represent the space around the body and the individual parts of the body that can be reached with the hands, the brain must, in particular, calculate the position of the arms in space (Kakei et al., 2003). Neuroscientific studies suggest that such a representation can be instantiated in a variety of different reference frames, relative to the eye's reference frame, with respect to the hand's reference frame, or with respect to the reference frame of an arbitrary point between these two (Gross and Graziano, 1995; Mullette-Gillman et al., 2009; Chang and Snyder, 2010; Galati et al., 2010; McGuire and Sabes, 2011). The term “reference frame” (RF) is used to refer to the center of a coordinate system to represent objects, including the body itself, and the relationships between objects (Cohen and Andersen, 2002).

In the study of peripersonal space, Rizzolatti et al. (1997) found that there are bimodal neurons that respond to the tactile stimulus on a limb but also to visual stimuli near this body part, regardless of the location of the limb in space and its posture. In addition, Làdavas (2002) established psychophysical evidence of how the visual perception of the peripersonal space is modulated by the motor representations acquired during the execution of the action.

In macaque monkeys, the posterior parietal cortex (PPC) is involved in the integration of multimodal information to construct a spatial representation of the outside world (relative to the body of the macaque or parts of it) to planning and the execution of object-centered movements (Sakata et al., 1995; Andersen, 1997; Murata et al., 2016). In particular, the intraparietal sulcus (IPS) serves as interfaces between perceptual and motor systems to control the movement of arms and eyes in space. Observations have shown that multimodal integration in these areas is based on a multiplicative integration, i.e., gain-modulation or gain-field (GF) mechanism (Andersen et al., 1985; Pouget and Sejnowski, 1997; Salinas and Thier, 2000; Salinas and Sejnowski, 2001; Blohm and Crawford, 2009). For example, Bremner and Andersen (2012) have proposed that gain-field neurons compute a fixation-centered reference frame by subtracting the vector between the eye location and the hand position to derive the hand position relative to the target in a reference frame centered on the eye; (see also Baraduc et al., 2001; Ustun, 2016). Nonetheless, the details of how these steps can be processed by parietal neurons using tactile input and how spatial transformation can be processed in a real physical system have never been expressed nor explained in earlier works. Particularly, most modeling works have assumed to know the location of hand in the visual space and the visual shape of the arm configuration. It is noteworthy that roboticists have started to consider this research problem for robots as we will present it further.

The details of this gain-modulation mechanism will be presented in section 2, but in order to have a better understanding of how it works, we present the data recorded by Bremner and Andersen (2012) of PPC units when a macaque performs a reaching task. The authors found that area 5d encodes the position of the hand relative to the eye before the presentation of the target to be grasped. But just after the presentation of the target, these neurons were sensitive to the location of the target relative to the position of the hand independent of the position of the hand or target locations as well as the direction of the eye gaze. That is, the most relevant information for a successful task was the location of the target relative to the hand as soon as the target is presented. Moreover, this representation is dynamic and constructed during the approach of the hand toward the target. This mechanism is particularly interesting in terms of computational efficiency, because not all the spatial combinations between the hand, the eye and the target are necessary to be learned for estimating novel and unseen relative locations.

**Figure 1:**
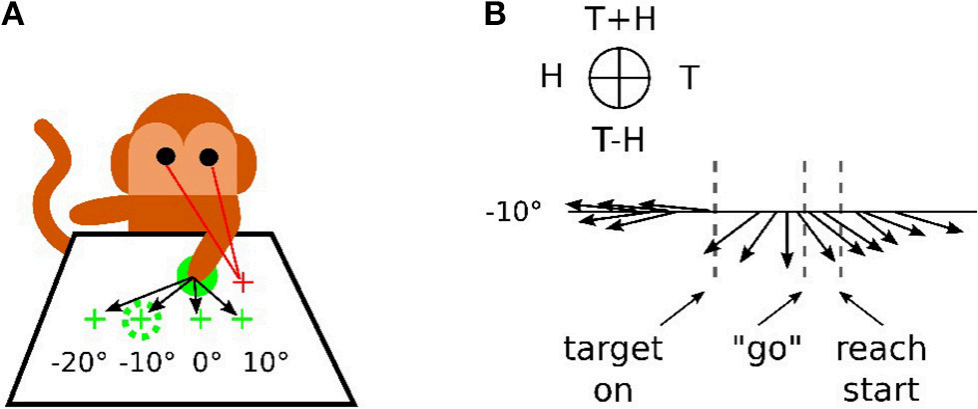
Recording responses of the PPC neuronal population in macaques. **(A)** Experimental setup for a reaching task. The eye fixation (red crosses), the initial position of the hand (plain green circle) and the targets (dashed green circles) were located at −20°, −10°, 0° or +10° horizontally. **(B)** response of the neuronal population for the target at −10° only, revealing the evolution of the mixed reference frames encoded during the task. The figures are adapted and reproduced from Bremner and Andersen (2014) from Figures 1, 4.

In Figure 1, we reproduce an excerpt of this work by Bremner and Andersen (2014) for a reaching task with different locations of the Target *T*, of the Eye *E* and of the relative distance to the Hand *H*. The Target location in Eye coordinates is denoted as (*T*) and Hand location in Eye coordinates is denoted as (*H*), whereas the location of Target-in-Hand coordinates is denoted as (*T*−*H*) and its opposite direction is denoted as (*T*+*H*). In Figure 1A, the eye fixation is expressed with the red cross located at +10° horizontally, the initial position of the hand is visualized with the plain green circle and the targets are shown with green crosses and the dashed green circle. In this work, Bremner and Andersen (2014) performed an analysis of the neuronal population response for different coordinate systems (Target-Eye, Target-Hand, Hand-Eye) oriented in three directions of a pie chart. Bremner and Anderson made the single-unit recordings from the posterior portion of dorsal area 5 (area 5d), in the surface cortex adjacent to the medial bank of the intraparietal sulcus (IPS). Recorded neural activity was passed through a headstage, then filtered, amplified, and digitized and saved for off-line sorting and analysis. As for the analysis, they used a gradient analysis to determine which variable within a pair [Target-Hand (TH), target-Eye (TE), or Hand-Eye (HE)] exerted the most influence on the firing rate of a cell, or whether both had equivalent influence. In conjunction with a gradient analysis, Bremner and Anderson used an SVD (Singular Value Decomposition) analysis to assess whether the relationship between pairs of variables was separable (in other words, a multiplicative, gain relationship) or inseparable (vector relationship). They also realized a time-step analysis to calculate the resultant length and angle of the coordinate framework gradient for each cell. Figure 1B presents the evolution of one neuronal population response for the target location at −10°. The pie chart at the top indicates the proper interpretation of the direction of the arrow for the pair of variables considered. The length of the arrow indicates the activity level and the orientation of the arrow indicates the sensitivity to one coordinate system. We can see from the graph that before the presentation of the target, the neuronal population codes the position of the hand *relative* to the eye gaze (H on the circular diagram at the top). When the target is presented, however, this population changes to code the location of the target relative to the hand (T-H on the pie chart).This result indicates the flexibility of parietal neurons to change the coordinate system dynamically to represent one spatial information. This is in line with recent observations of parietal neurons found sensitive to different spatial coordinates centered in the shoulder RF, the elbow or a mixture of them with respect to the context; a phenomenon referred as non-linear-mixed selectivity to designate this dynamic calculation made by parietal neurons (Zhang et al., 2017). The gain-field mechanism is one of few computational mechanisms that can support these types of dynamical transformation necessary for spatial representation by fusing the What and Where pathways.

In robotics, Hoffmann et al. (2010) presented one of the rare states of the art on the body schema from the perspective of robotics. Most of the review was focused on integrating visual and proprioceptive information. For instance, the better part of the robotic experiments were designed in using the linear combination of basic functions for visuomotor transformations (Halgand et al., 2010; Chinellato et al., 2011; Schillaci et al., 2014). However, in these works, the tactile information was not considered at all and it would have been interesting to use an artificial skin to contribute to the representation of the body schema and its space around as an additional modality with respect to the visual and proprioceptive modalities.

Hikita et al. (2008) proposed a bio-inspired model of the body representation of the robot through these three modalities. They used tactile information to trigger a Hebbian learning to associate the position of the arm with the focus point of visual attention when the robot touches the target with its hand or with a tool. This model allows taking into account the behavior of parietal bimodal neurons observed by Iriki et al. (1996).

The work of Roncone et al. (2015) also focuses on representation body and peripersonal space using an artificial skin. They concede, however, that their approach relies instead on existing engineering solutions and targets practical functionalities compared to the studies presented by Hikita et al. (2008). They associated each touch unit with a spatial receptive field extending in 3D space around the surface of the skin. Stimulations in the form of motor or visual events are detected and recorded. The developed architecture estimates the probability of contact with anyone which part of the body, i.e., to predict the tactile contact and to adapt the robot behavior to avoid or grasp an object (Roncone et al., 2016).

More recently, robotics studies with artificial skin have been developed to investigate biologically motivated models of peripersonal space. For instance, Roncone et al. (2016), Hoffmann et al. (2017) focused on the topological organization of visuo-tactile receptive fields in cortical maps to organize actions for an avoidance or reaching movement. Born et al. (2017) proposed a model of invariance learning based on Hebb's rule for the development of hand-centered visual representations. Lanillos et al. (2017) instead emphasized a predictive coding approach for discovering causal relationships in visual, tactile and motor stream to discriminate ego-motion and body parts.

In this paper, we propose a neural architecture of body and peripersonal space representation that relies on the integration of multiple feedbacks from the robot body; i.e., its proprioception, its tactile input and its vision. Our contributions are in the use of (1) the mechanism of gain-field neuromodulation as a main mechanism for integrating modalities from different reference frames and (2) an artificial skin developed for a robotic purpose. The model developed allows rebuilding the location of the arm in the visual field and the location of objects relative to the somatosensory field by aligning the different modalities from each other. Most importantly, the results obtained are close to the behavior of the parietal neurons recorded in the parietal cortex area 5d, presented in the work of Bremner and Andersen (2014): in comparison with the Bremner's and Anderson's work, our architecture allows us to represent the object location relative to the moving arm as soon as the object is presented by combining proprioceptive, visual and tactile inputs from the three different reference frames. And this representation is dynamic and constructed during the approach of the hand toward the target. We will present two robotic experiments with a similar protocol in sections 3.2 and 3.3.

Our experiments can contribute to the understanding of the biological principle of the peripersonal space representation. In this respect, they reinforce our previous works on spatial representation (Pitti et al., 2012, 2017; Mahé et al., 2015; Abrossimoff et al., 2018).

## 2. Materials and Methods

### 2.1. Material

**Figure 2:**
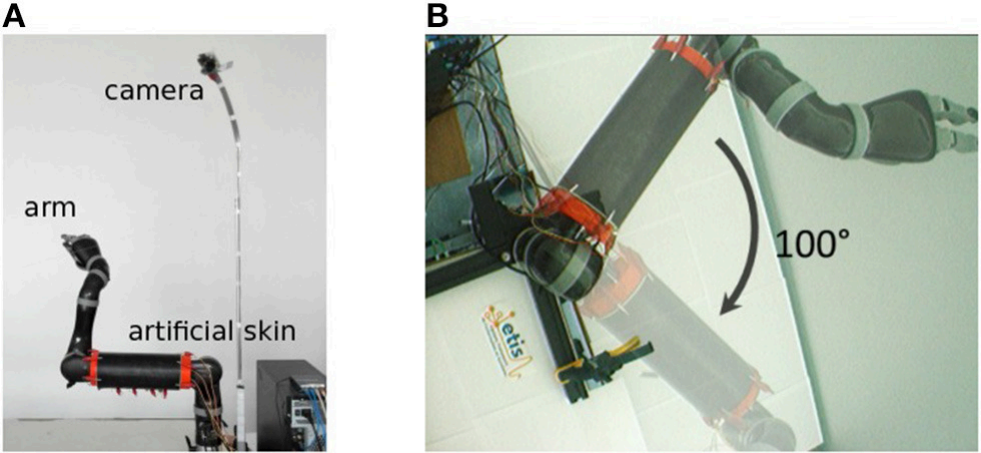
Experimental setup used in our experiments. **(A)** Robot arm covered with the artificial skin and firewire camera fixed in height. **(B)** Visual field of the camera and aperture angle of 100° of the robot arm.

In our experiments, we use the Jaco robot arm from Kinova covered with an artificial skin that we developed, its properties are extensively presented elsewhere in Pugach et al. (2013, 2015, 2016). The visual input is commonly acquired by a static firewire camera fixed in height so that it can view the full arm moving, see Figure 2.

#### 2.1.1. Artificial Skin

The artificial skin is a rectangular conductive fabric of dimension 250 × 320 mm with sixteen electrodes attached uniformly along the perimeter. The fabric resistance decreases when pressured. We use it in our previous works in order to develop a low-cost system based on the Electrical Impedance Tomography method (EIT) for data acquisition from the conductive fabric. The EIT is a non-invasive technique particularly used in medical imaging to reconstruct an internal spatial distribution of conductivity/resistivity from measuring iteratively the voltages from different current locations through electrodes placed on the circumference of the investigated object. The electronic hardware and the neural reconstruction are detailed in Pugach et al. (2013, 2015) and a touch-based control of the Jaco Arm covered with our artificial skin is detailed in Pugach et al. (2016). The spatial patterns of the tactile contact can be acquired and localized at a frequency of 40 Hz.

#### 2.1.2. Vision System

The camera provides a video stream of 30 frames per second and a resolution of 160 by 120 pixels. The arm is in the center of the camera visual field. For the sake of simplicity, we have limited the arm to a single degree of freedom in the visual plane of the camera. The maximum angle of joint movement is 100°.

### 2.2. Methods

#### 2.2.1. Gain-Field Mechanism

**Figure 3:**
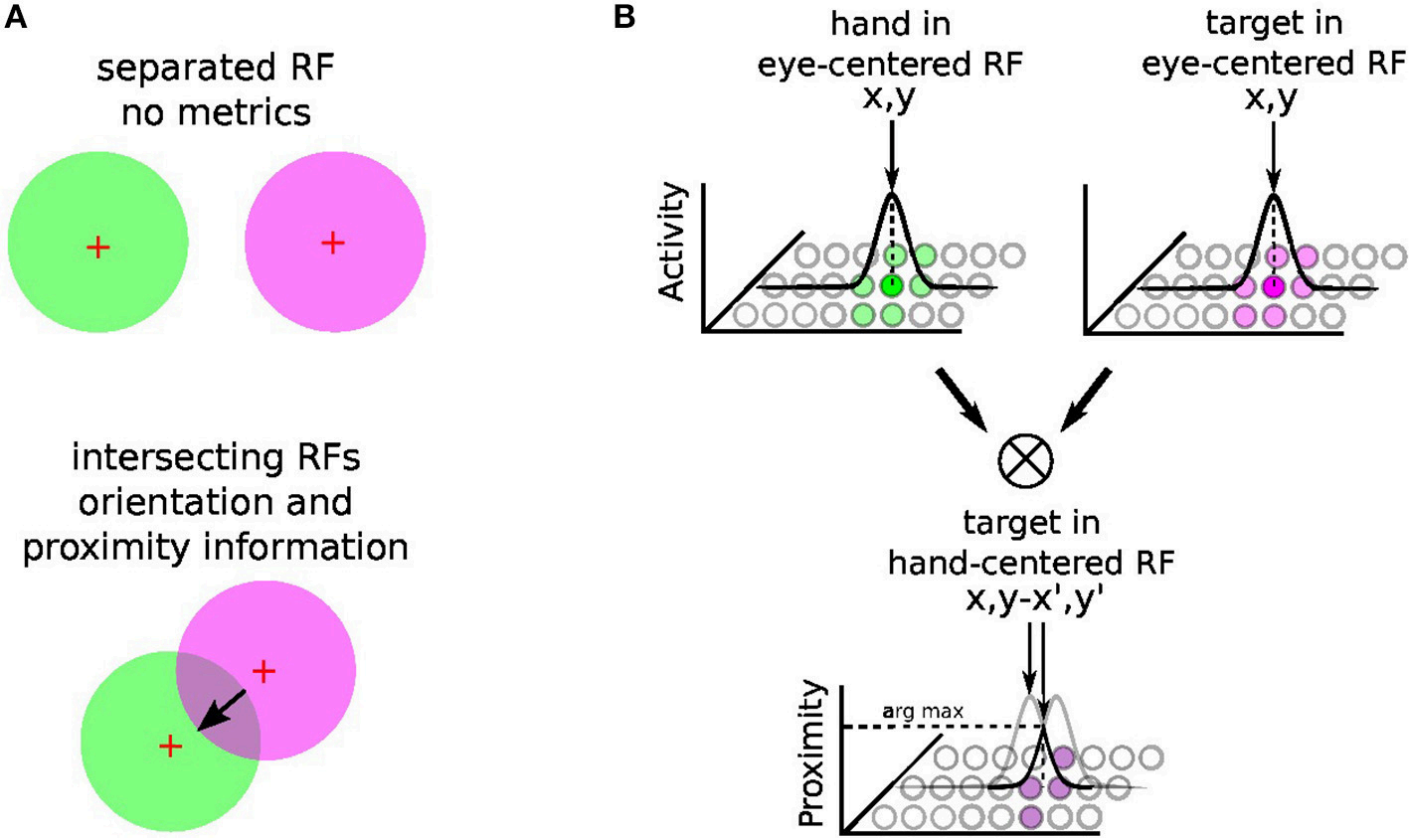
Gain-field mechanism for spatial transformation and hand-centered representation. The mechanism of gain-field modulation discovered in parietal neurons corresponds to a multiplicative interaction across signals of different modalities (e.g., visual, tactile, auditive, or proprioceptive). Gain-field neurons see their amplitude level to vary with respect to targets motion relative to different reference frames, which can be arm-, head-, hand- or eye-centered. The multiplicative property between two receptive fields can permit the spatial transformation from one reference frame to another with a resulting receptive field whose amplitude and orientation relates directly to it; see **(A)**. For reaching one target, GF neurons may construct one arm-centered receptives fields to represent the distance and orientation to the target, presumably from the multiplication between hand and targets seen visually in the eye-centered RF; see **(B)**. Hypothesis reproduced from Ustun (2016) and Chang and Snyder (2010).

The principle of integration behind gain-field neurons for spatial transformation is based on the by-product of the neural fields' activity between two or more modalities (Blohm and Crawford, 2009; Ustun, 2016); e.g., *X* and *Y* modalities. For instance, Figure 3 shows the multiplicative binding *X* × *Y* between two neural fields *X* and *Y*, which can serve then to construct a relative metric to transpose signals from one reference frame to another. The amplitude level of the resulting neural field indicates their vicinity whereas its shape indicates their relative orientation (arrow). Such computation is similar to sigma-pi networks or radial basis functions networks and has been rediscovered recently in computer vision as gated networks for categorizing transformations (Memisevic, 2011). In robotics, gated networks have been emphasized recently by Sigaud et al. (2016), Sigaud and Droniou (2016), and Memisevic (2010) but they have been used mostly for categorization and not for spatial transformation as performed by gain-field networks–, for which the activity of each unit is meaningful and corresponds to a metric value and not a label.

In our case, gain-field networks will serve for two computations: learning where the arm is in the eye field—e.g., eye-centered RF, combining touch, visual and proprioceptive information—and learning where the target is relative to the arm (e.g., arm-centered RF); see Figure 3B. We explain first the mechanism of gain modulation and its equation in the next section 2.2.2, we present then in details how spatial transformation is done in the case of arm reaching in section 2.2.3.

#### 2.2.2. Gain-Field Networks

Gated or gain-modulated networks are an instance of sigma-pi networks constituted of radial basis functions pre-defined parametrically or learned that produce a weighted sum of joint probability distributions as output (Pouget and Sejnowski, 1997). The output terms *Z* are a linear combination of the product of the input variables *X* and *Y* whose cardinalities are respectively *n*_*Z*_, *n*_*X*_ and *n*_*Y*_, so that predicting Ẑ consists on computing for all values *Z*_*k*_ of *Z*, *k* ∈ *n*_*Z*_:

(1) $$\begin{array}{r} \forall k,Z_{k}=\overset{n_{X}}{\underset{i}{\sum }}\overset{n_{Y}}{\underset{j}{\sum }}W_{ijk}\left(X_{i}\times Y_{j}\right), \end{array}$$

with *W* synaptic coefficients in *n*_*X*_ × *n*_*Y*_ × *n*_*Z*_. Since this matrix can be quite large, a way to reduce drastically the dimensionality of the gain-field networks is to multiply term by term, each *X*_*i*_ and *Y*_*i*_ with *i* ∈ *n*_*X*_, but this is not done in this work.

The global error *E* is defined as the Euclidean distance calculated between *Z* and Ẑ for all the input examples. The optimization function used for learning the synaptic weights of the output layer *Z* is the classical stochastic descent gradient. This is in line with our previous works (Pitti et al., 2012; Mahé et al., 2015; Abrossimoff et al., 2018), and differs slightly from Memisevic (2011) as they applied the algorithm to image problems only, not to robotics.

#### 2.2.3. Neural Architecture for Spatial Representation

Using the gain-field mechanism presented earlier, it is possible then to exploit their computational capabilities to represent the arm in the visual field (i.e., the body schema) as well as the location of the target relative to the arm (i.e., the peripersonal space).

**Figure 4:**
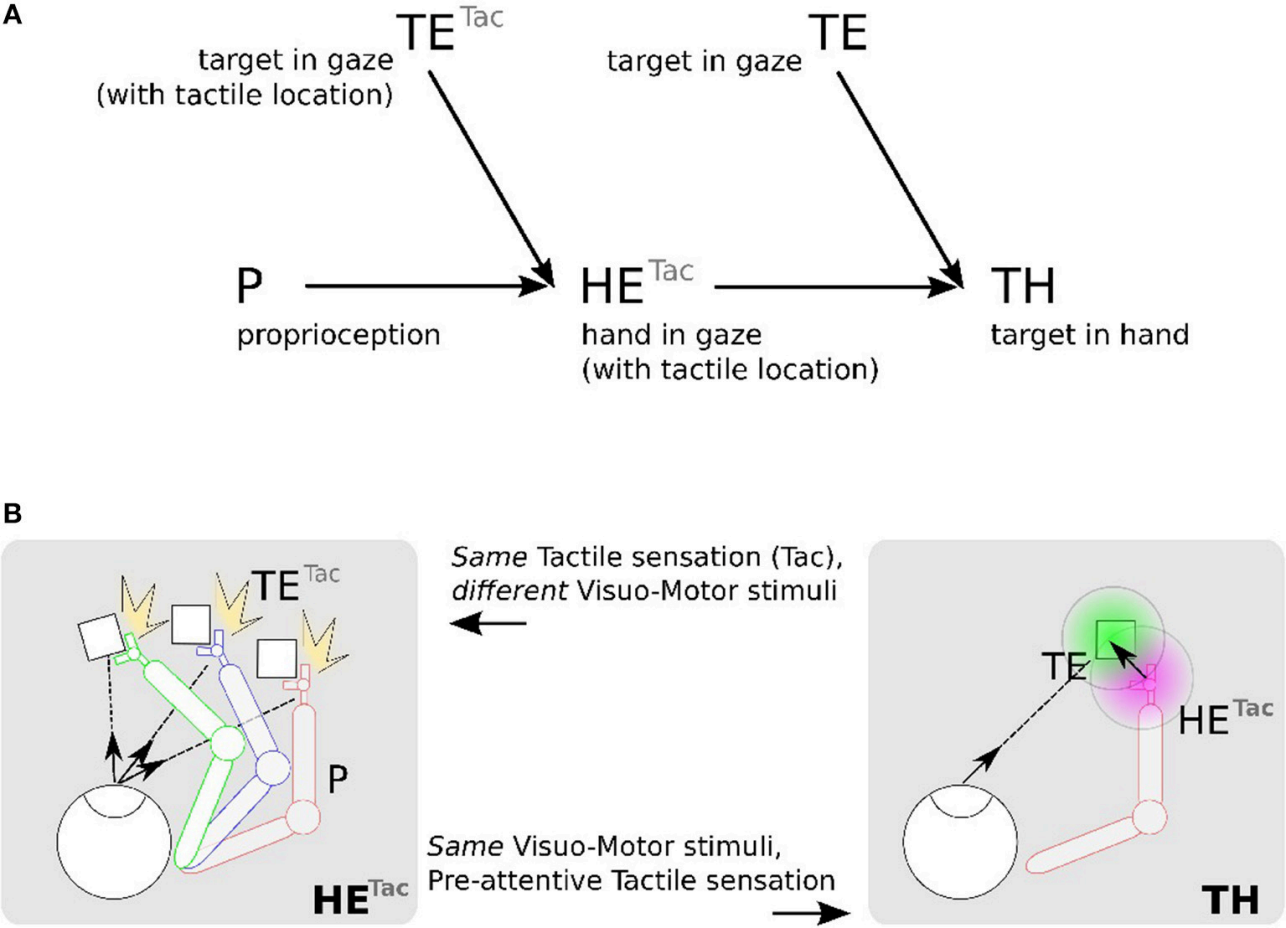
Proposed mechanism for constructing a hand-centered represention from multimodal infomation. **(A)** We can construct in a hand-centered reference frame (TH) by merging the hand location (HE) and target location in the eye-centered visual space (TE). Locating the hand in the visual space (HE) can be done by binding the location of seen targets (TE) and the proprioceptive information of the arm posture (P) *conditionally* to the perceived tactile sensation ^*Tac*^. **(B)**. *TE*^*Tac*^ and *HE*^*Tac*^ can be constructed from multiple associations between visuomotor pairs only when a contact occurs (left). From the predicted location of the hand from *HE*^*Tac*^, it possible then to estimate visually the distance and orientation to the hand (right).

Figure 4A shows this computational process decomposed into three steps: (1) location of the hand (tactile information) in the eye field from visuo-motor integration (Hand in Eye), (2) location of the target in the visual field (Target in Eye), (3) detection of the target position relative to the robotic arm (Target in Hand). We make the note that in this figure the eye is fixed and only the arm is moving.

We detail now the implementation steps of our computational model. The first part aims at learning the spatial location of the arm in the visual reference frame from the tactile input, see Figures 4A,B in the left figures. Here, various experiences of tactile feedback for different visual target position and motor/proprioceptive configuration permit to learn the visual location of a ‘touched' target together with the arm configuration (the motor angle); explanation in section 2.2.4.1. This stage permits to build a visual reference frame centered on the arm. The second part aims at estimating the relative distance in the visual field between the arm-centered RF computed previously and the target RF, see Figures 4A,B on the right figures. This will permit to compute peripersonal space and pre-attentive tactile sensation.

#### 2.2.4. Implementation

For simplicity, the vision system is based on color recognition. The input image is in RGB format and 160 x 120 pixels resolution. This image is first converted to HSV (Hue Saturation Value) in order to retrieve exemplars of which we vary the Hue. These variations make it possible to extract the predominance of a chosen color within the image. We then perform a binarization of the image, the initial image is transformed into a black and white image where all the pixels have only two values 0 and 1. We project later this image on neural fields of the same dimension.

##### 2.2.4.1. Part 1, arm in the eye-centered RF (HE)

**Figure 5:**
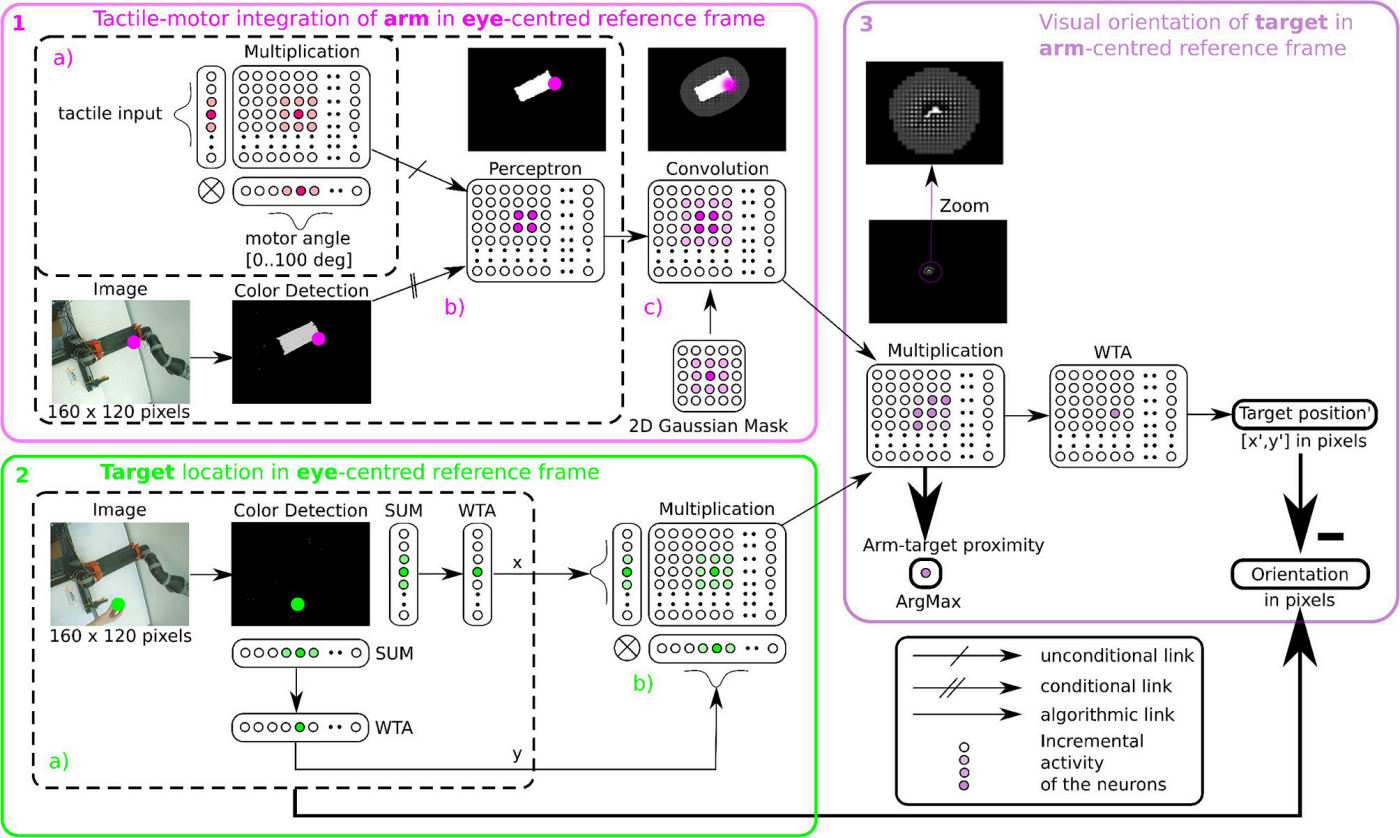
Algorithmic implementation of tactile, visual, and motor integration. The principle of integration is based on the three successive step marked in pink **(1)**, green **(2)**, and purple **(3)**. Descriptions of each part are presented in section 2.2.4.1 respectively. Part 1 and 2 correspond to tactile-motor integration based on GF computation for eye-centered RF of the arm (HE) and of the target (TE); they contain subparts **(a–c)** outlined in dotted lines. Part 3 corresponds to the computation of the target in arm-centered RF (TH).

In order to determine where the arm is in the visual space, we use tactile information as a conditioning signal to combine proprioceptive information and visual information as explained in the previous section, see Figure 5-1a Tactile input modulates the learning rate as a “Go signal,” meaning that no tactile input induces no learning at all.

The learning stage is done using Equation (1) to associate the tactile and proprioceptive information to visual information, see Figure 5-1b. We fix the arm in an angular position and touch the artificial skin with an object (the focal point of the visual attention). Whenever the object touches the arm, the visual neuron associated with the tactile receptive field learns the combination of the touched visual position with the angular configuration of the arm / joint. Note that in the case of a bimanual robot, we may achieve tactile self-stimulation and thus provide self-calibration of the robotic body with artificial skin.

Recall that the learning algorithm of a neural network with Perceptron units consists in modifying synaptic weights *W* until finding the minimum mean squared error between the input *X* (i.e., the joint distribution between the motor angle and the tactile input) and the desired output *D* (i.e., the visual location of the target on the robotic arm). The equations of the learning rule and the output of each neuron are the same as the ones presented in section 2.2.2.

Furthermore, in order to model a spatial receptive field around the arm, i.e., the peripersonal space, we apply a Gaussian 2D mask on the output network (see Figure 5-1c). This mathematical operation permits to create a soft and smooth outline around the arm.

##### 2.2.4.2. Part 2, target in the eye-centered RF (TE)

After having learned the representation of the robotic arm in the visual field (HE), we use the simpler attention mechanism exploiting visual information only to represent the target in the eye-centered reference frame (TE). The determination of the position of the target is based on color recognition. An RGB image of the same size 160 × 120 pixels is converted to HSV and is subsequently binarized in correspondence with the color of the object. Thereafter, we project this binarized image on a neural field of the same dimension. Finally, we locate the *x* and *y* coordinates of the object's center in the visual field after selecting the most active neural position (see Figure 5-2.2a). This competition is made through a Winner Takes All rule (WTA) (Rumelhart and Zipser, 1985; Carpenter and Grossberg, 1988). The winning neuron generates an output at 1, the other neurons are set to 0. The target representation in eye-centered RF is performed by multiplicative neurons, the multiplication of WTA vectors with a Gaussian curve centered on *x* and *y* (see Figure 5-2b).

##### 2.2.4.3. Part 3, target in the arm-centered RF (TH)

Once we have processed the position of the target and of the arm in the visual field, it is possible to compute their relative distance using the gain-field framework as presented in Figures 3, 4, which corresponds to the third part in Figure 5. This final layer is similar to the previous layers using basis functions. The product between two neural fields, the neurons perform a mutual information encoding between the two modalities, i.e., between the reference centered on the arm and the repository centered on the target. To derive the location of the target relative to the hand, we subtract the vectors between the target location on the eye (position *x*, *y* of the focal point of attention, cf Figure 5-2a) and the mutual center point (the coordinates *x*′, *y*′ defined by WTA). The proximity of the target to the arm is defined by the amplitude level of the mutual center point taken from the *argmax* function (see Figure 3) and is converted to a value between 0 and 1. A value of 0 indicates that the target is far from the arm and is not in the peripersonal space. The value of 1 indicates that the target is touching the arm, which is confirmed by tactile feedback.

## 3. Results

In this section, we present the results of three experiments using the proposed model of tactile, visual and proprioceptive integration to represent the body schema and the peripersonal space. The purpose of the first experiment is to present how the neural architecture represents the space around the body centered on the arm. The second and third experiments aim at modeling the similar behaviors of parietal neurons for coding information about the arm in the visual space and about the target in the arm-centered reference frame.

### 3.1. Experiment 1 - Representation of Space Around the Body

As explained in section 2.2.4, the first part of the learning stage consists of associating the proprioceptive information of the robotic arm with the visual location of a target in order to reconstruct its visual mapping. This is done for various arm configurations with tactile information as a conditioning signal for calibration.

We make the remark that it is possible to not use tactile input for the visual reconstruction as we have done in Abrossimoff et al. (2018), but without tactile information, the learning phase can take a long time because there is a very large number of possible combinations between the pixel values and the angular positions of the arm. Using tactile information instead, it can make this phase easier by making the correspondence between the visual location of one stimulus on the artificial skin and the spatial configuration of the arm, only when touched. Each motor angle is discretized in 100 units by population coding with a Gaussian kernel centered on the current motor angle. We record the activity of the visual neural network for all angles of the motor conditionally to the tactile activity.

After the learning phase, the output neurons from network Figure 5-1.1b are able to predict the visual representation of the arm even if the tactile information is not provided. The visual representation of the tactile units can be simply retrieved back from the learned model if we activate all the tactile units in the network Figure 5-1a. By doing so, it is possible to estimate the spatial distribution of all the receptive fields of the tactile units; which means, we can reconstruct the spatial location of the whole arm in the visual scene while loosing the information of each RF.

**Figure 6:**
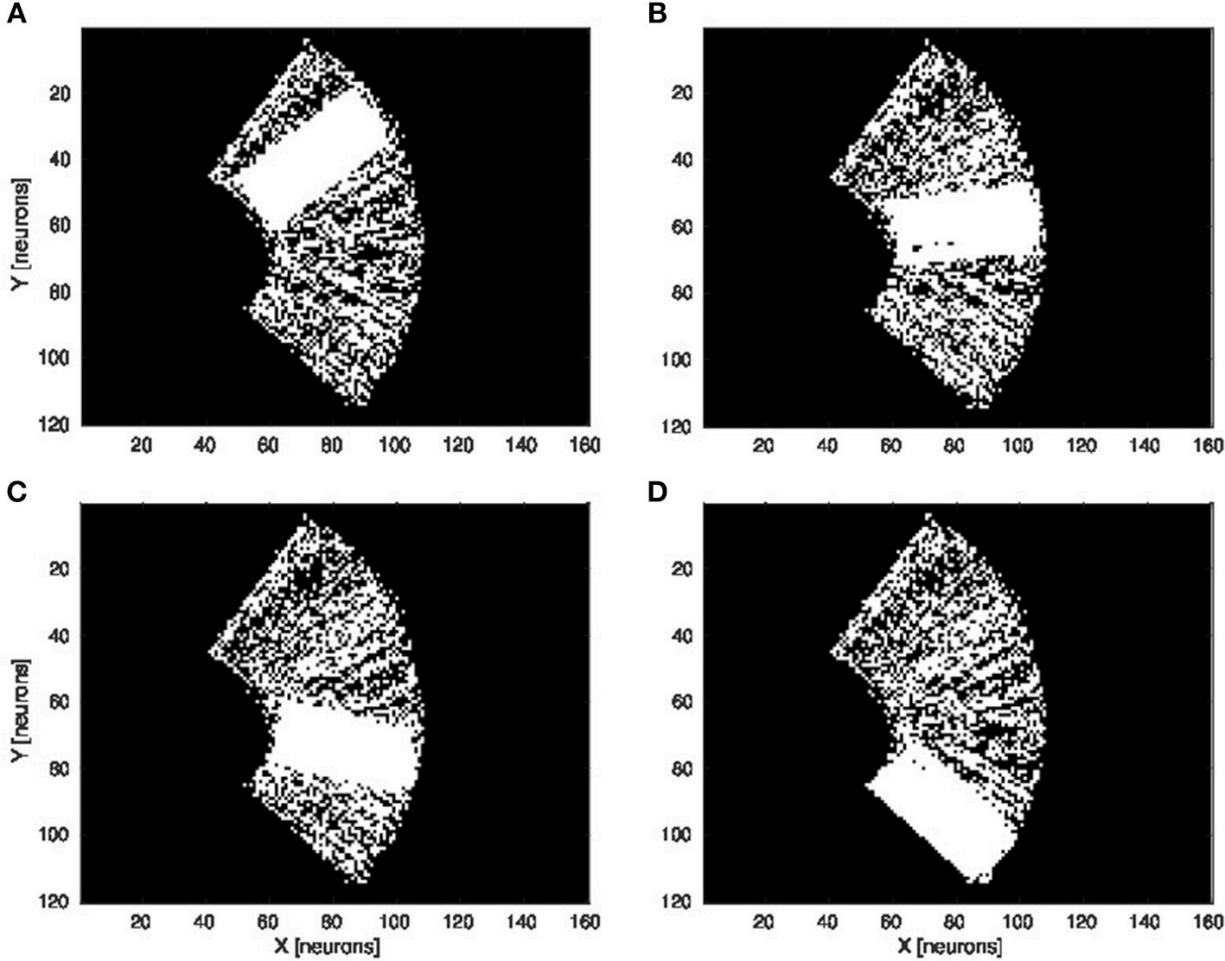
Visual prediction of the complete tactile RF distribution and of the whole arm location. Visual perceptron units estimate the visual location of each tactile-motor GF unit (i.e., the body schema). The learning is done only when a visual target touches the arm and depends on the specific tactile location on the artificial skin and on the specific arm motor angle. By activating virtually all the tactile units and for a specific motor configuration, it is possible to display the density distribution of the arm location in the visual field. The results are presented for four different motor positions: 20°, 50°, 70°, and 100°, respectively **(A–D)**.

**Figure 7:**
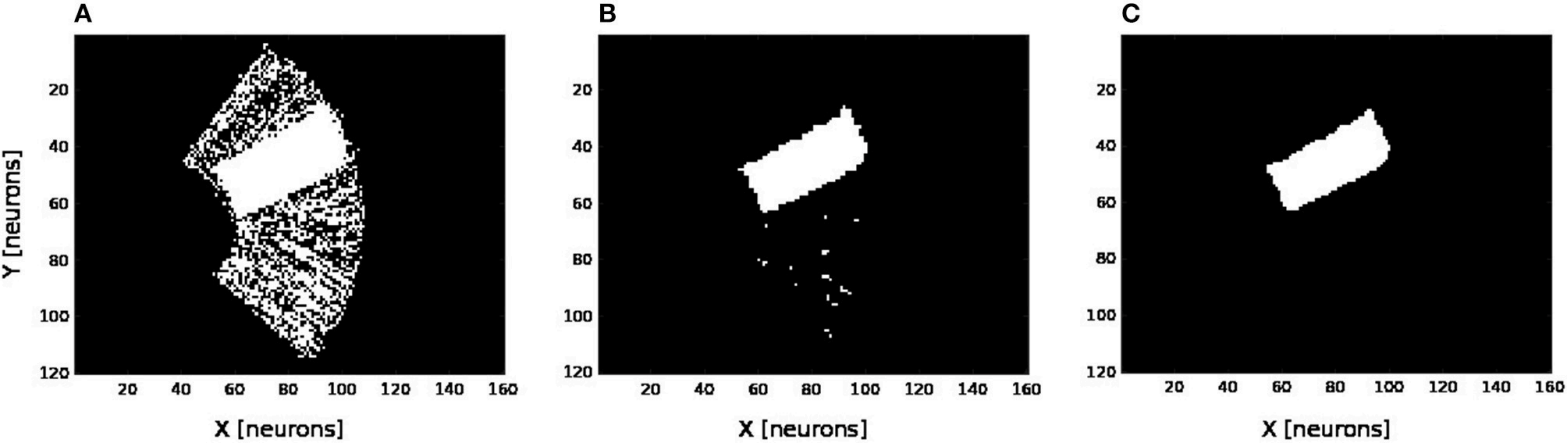
Noise filtering operation. After the visual prediction of the whole arm location as showed in Figure 6, we add a filtering operation to denoise the erronous and isolated units till having a uniform density distribution; for the motor angle 30°. **(A)** Before denoising. **(B,C)** After denoising in two stages.

We present in Figures 6A–D the estimation of the full-arm posture after the learning stage for four different motor angles, 20°, 50°, 70°, and 100°. We can observe that the estimation, although noisy, represents well the arm configuration, although for a simple transformation like a rotation. In order to eliminate the noise of the spatial density distribution of the arm location, we applied a mean-field filter and then used a binary thresholding of the neurons twice, see Figure 7. In image processing, the mean filter is defined as the average of all pixels within a local region of an image. The same process is done with neural populations. Neurons that are included in the averaging operation are specified by a mask. As a first step, we have used a larger filtering mask to remove big tailed noise and as a second step, we have used then a smaller filtering mask to remove small noise. This may exempt to using vision to determine the position of the arm in the visual field when the arm is occluded or in the dark or to determine the relative distance of multiple locations on the arm (e.g., hand, forearm, elbow) to the target.

**Figure 8:**
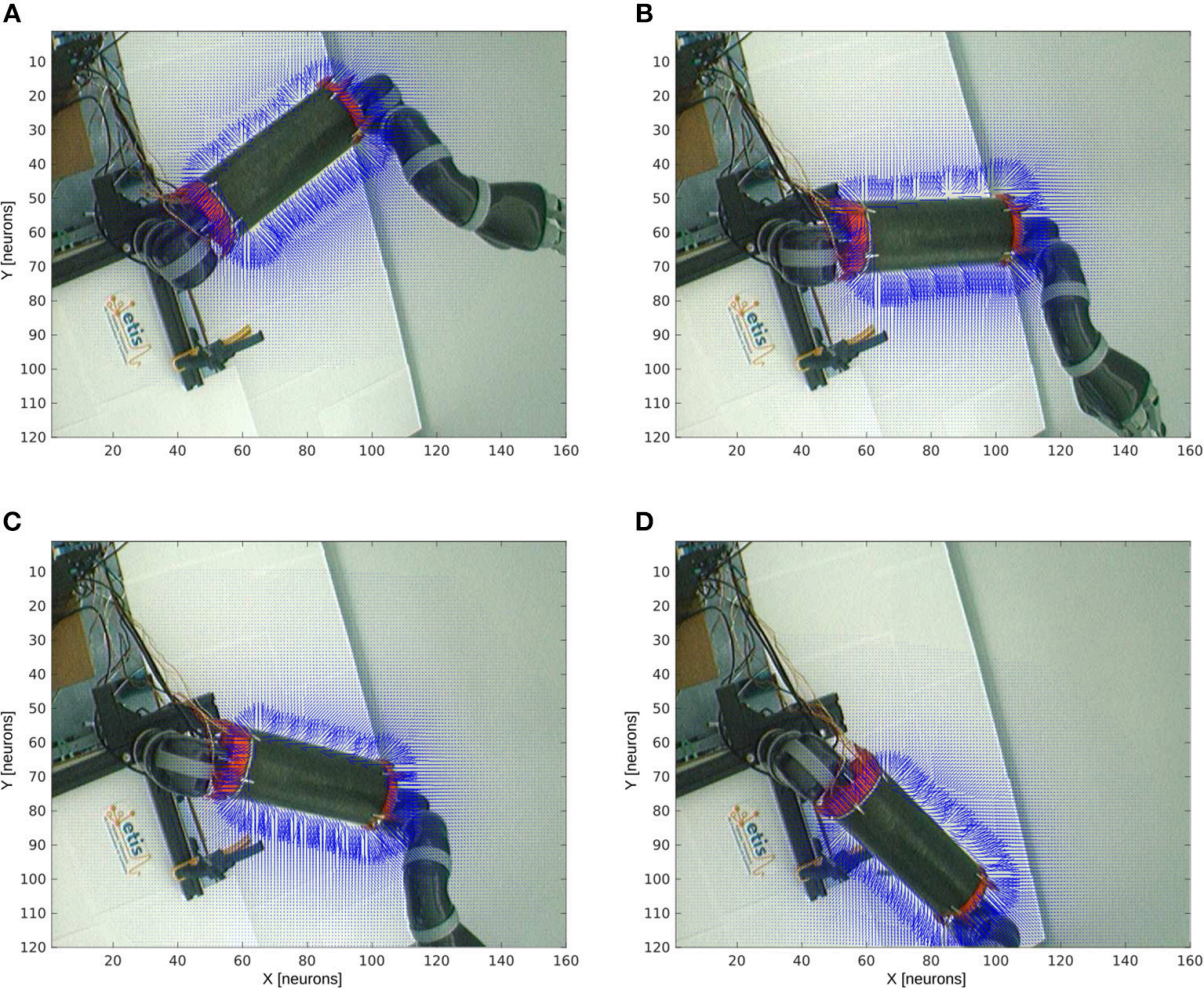
Peripersonal space and arm-centered RF for various motor positions. We display in **(A–D)** the information about the compound GF receptive fields resulting from the interaction between the hand and target receptive fields, for all the target locations in the visual space and for the four motor angles 20°, 50°, 70°, and 100° respectively. Each vector indicates the orientation of the compound GF receptive field and their length indicates the proximity of the target to the robot arm. The direction of the arrows changes with respect to the motor angle and their length is non-linearly proportional to the distance to it.

Figure 8 shows the receptive fields of the visuo-tactile neurons computed for four different positions of the robotic arm and for all the locations of the target in the visual space; see the output network of Figure 5-3. This image has been obtained by collecting the spatial orientation and distance between the skin and the target computed from the neurons activity from the output network. For all the visual positions of the target around the arm, an arrow has been projected proportional to the amplitude level of the neural field and in the direction of the target as explained in Figures 4, 5-3.

Without any target nearby the arm, the receptive fields aim at representing where the arm is. In the presence of a target within reach, however, the receptive fields serve to compute where the target is relative to the arm. This property of body representation has been observed by Graziano and Aflalo (2007).

With respect to the distance to the arm, the neural activity that computes the receptive fields is non-linear: the activity of the cells is higher when a target is placed nearby the skin while it decreases following a power-law scale when the distance to the arm augments. This is a consequence of the two gaussian field's multiplication. Thus, the more a target is entering the receptive fields, the more they encode with better precision its spatial distance and orientation. They are therefore more sensitive to nearby objects.

Furthermore, we can see also that our architecture is able to correctly predict the body schema as well as to represent its peripersonal space with respect to the arm position. This property of dynamic encoding has been observed for instance by Iriki et al. (1996).

### 3.2. Experiment 2 - Estimation of Visual Distance and Orientation of Target-Centered GF Neurons When the Arm Moves and the Target Is Fixed

**Figure 9:**
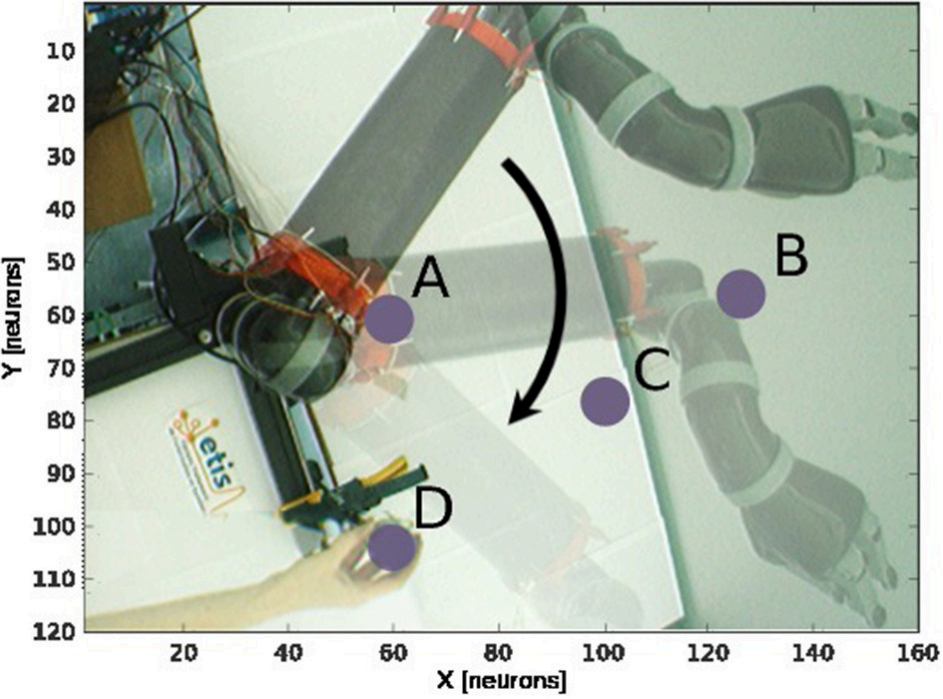
Experience 2 – Target-centered receptive field. Experience done for fixed targets in four locations **(A–D)** when the arm moves.

The second experiment aims at replicating Bremner and Andersen (2014) observation of hand-centered parietal neurons sensitive to the relative distance and orientation of the hand to target (in our case the arm). Their activity level depends on both the position of the arm (proprioception) and the location of the target in the eye field. We present in Figure 9 the scenario of the experiment. We set the target to four positions Figures 9A–D and we move the arm within the interval range between [0 and 100°]. Every 10°, we record the activity of the multiplicative neuron which performs the computation of the relative distance between the arm and the visual target as explained in Figure 4 and Figure 5-3.

**Figure 10:**
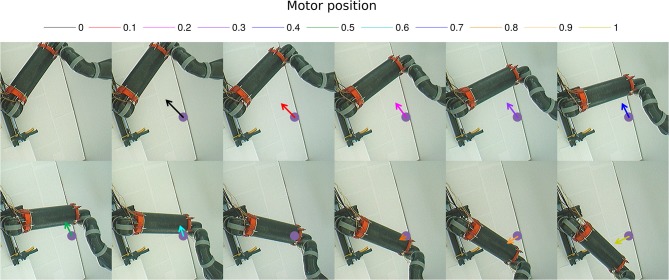
Gain-field visual unit receptive field centered on the target C. Snapshots of the relative visual distance and orientation for the target located in **C** in Figure 9 computed by the compound GF units from the arm and target centered RF when the arms moves in the interval [0°−100°]. The arrows length corresponds to the amplitude level of the neurons, i.e., the proximity of the RF, and their orientation corresponds to the orientation. The color code represents the different motor positions. For better visualization, we have increased the length of the arrow by 10 times.

**Figure 11:**
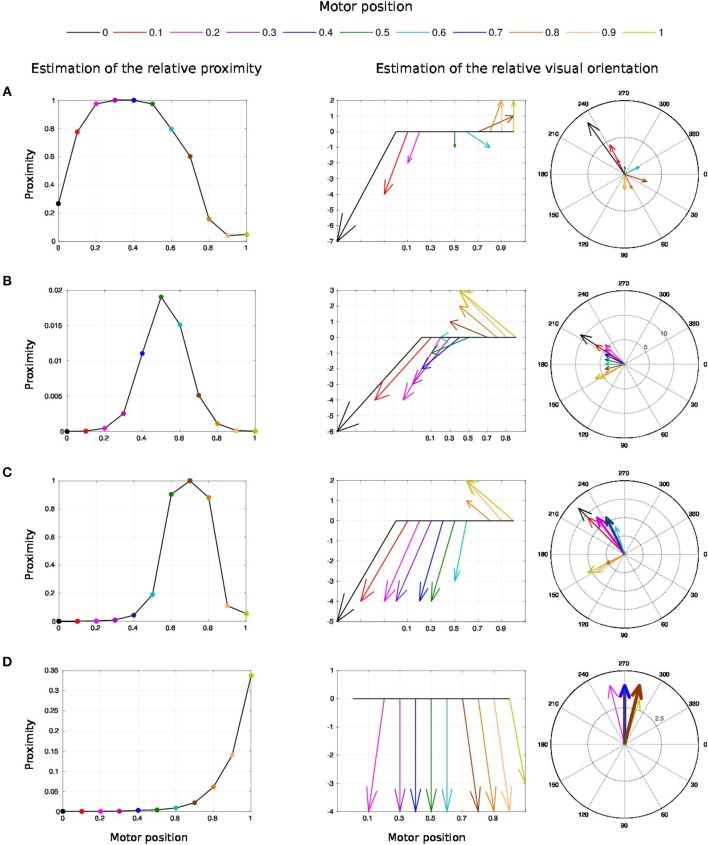
Spatial receptive fields and relative visual orientation of target-centered GF neurons. The left charts correspond to the estimated proximity of the four GF neurons target centered at fixed positions respectively at locations **(A–D)**. The y axis corresponds to amplitude level of the neurons whereas the x axis and the color code represent the different motor positions between [0 and 100°] and normalized between [0 and 1]. Location A is the nearest to the arm and location D is the farthest. Each target-centered neuron show different types of receptive field with respect to the distance to the arm. The higher the amplitude level is, the closer the arm is with respect to the target. When the amplitude level reaches 1, it indicates that the target is above the arm in the visual space. The middle and right charts represent the estimated relative visual orientation between the target and the robot arm. The middle plot displays orientation vectors coming from equally spaced points along a horizontal axis. It expresses the orientation vector components relative to the origin of the respective orientation vector. The x-axis and the color code represent the different motor positions, the arrows' length corresponds to the amplitude level of the neurons, i.e., the proximity of the RF, and their orientation corresponds to the orientation. The y-axis represents the y components in relative coordinates. The right chart presents the same information in polar coordinates centered at the target location.

We draw in Figure 10 the relative visual distance and orientation of the receptive field computed with respect to the target location **C** and the most active tactile neuron retrieved. The arm moves toward the target, touches it and goes beyond it. The length of the arrow indicates the sensitivity of the RF whereas its orientation points to the nearest tactile point. The details of the neural activity retrieved for the four locations are presented in Figure 11 and Supplemental Data. The left chart displays the amplitude level of the neuron taken from *argmax* function in resulting spatial RF between the arm and the target, which permits to have an estimation of the relative proximity. The middle displays the relative orientation angle in radian with respect to the motor position normalize between [0 and 1] and the right chart presents the same information in polar coordinates centered at the target location. The colors correspond to the angular motor positions. The length of the vector indicates the relative distance as in the previous experiment.

For location **A**, we observe that the target is in the peripersonal space during the entire movement of the arm and most of the time in an area of high activity. The multiplicative neuron encodes the location of the target in a mutual reference frame and changes between 0.05 and 1 (see Figure 11A). The maximum activity corresponds to the motor positions for 30° to 40°. This means that the focal point of attention is above the position of the artificial skin, which is confirmed by the orientation graphs.

In these graphs, the orientations for the arm positions 30° and 40° are missing. The neuron does not encode orientation for maximal activity because the target is within the visual location of the skin. We make the note that the experiment was organized so that the target did not touch the skin in order to have a stable visual response of the target's location.

For location **B**, the focal point of attention is quite far away from the arm, which corresponds to a weak activity of the neuron. The maximum activity does not exceed 0.02 but it is still possible to estimate the relative visual orientation from the resulting neural field. The neuronal activity varies in a narrow range (between 150 and 210°) relative to the previous location of the target. For location **C**, we find a small variation in the neural activity for motor positions from 0 to 50° because of the large relative distance. The contact with the skin coincides with the motor position at 70°. And for location **D**, we see that the orientation is absent from the initial position in Figure 11D. The neural activity is 0. This means that the focal point of attention is out of the peripersonal space. But once the neuron activity becomes different from zero, the relative orientation of the target can be retrieved even when the activity is very low and does not exceed 0.025.

As a short conclusion of this experiment, these results show that our neural architecture can encode information about relative proximity and orientation of a target with respect to the arm in a mutual reference frame. Neurons react independently of the location of the arm and of target in the visual field. The results obtained are therefore close to the recordings made by Bremner and Andersen (2014) of the parietal neurons in zone 5d.

### 3.3. Experiment 3 - Estimation of the Visual Distance and Orientation of Arm-Centered GF Neurons When the Target Moves to the Arm

The third experiment is the alternative version of experiment 2 expect that we fix the arm position to a certain location and move the targets toward it. The aim of experiment 2 was to analyze the change in estimating the relative distance and orientation of the arm toward targets during a reaching task. Besides, the aim of experiment 3 is to analyze the change in estimating the relative distance and orientation of approaching targets when the arm is fixed. It is not clear though whether the two experiments would give the same results, however this experiment aims at replicating the results of Graziano and Botvinick (2002) and Bremner and Andersen (2014) showing that the activity level of the parietal neurons depends on the position of the arm position (proprioception) and the location of the object in the visual field.

**Figure 12:**
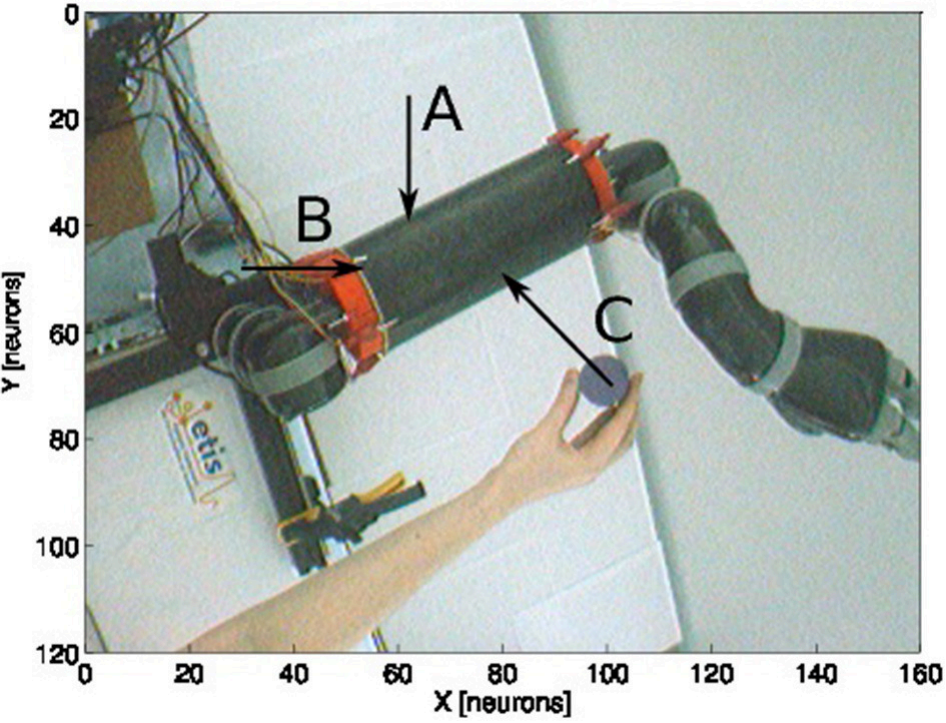
Experience 3 – Arm-centered receptive fields. Experience done for three looming targets at different locations **(A–C)** in the direction of the arm.

**Figure 13:**
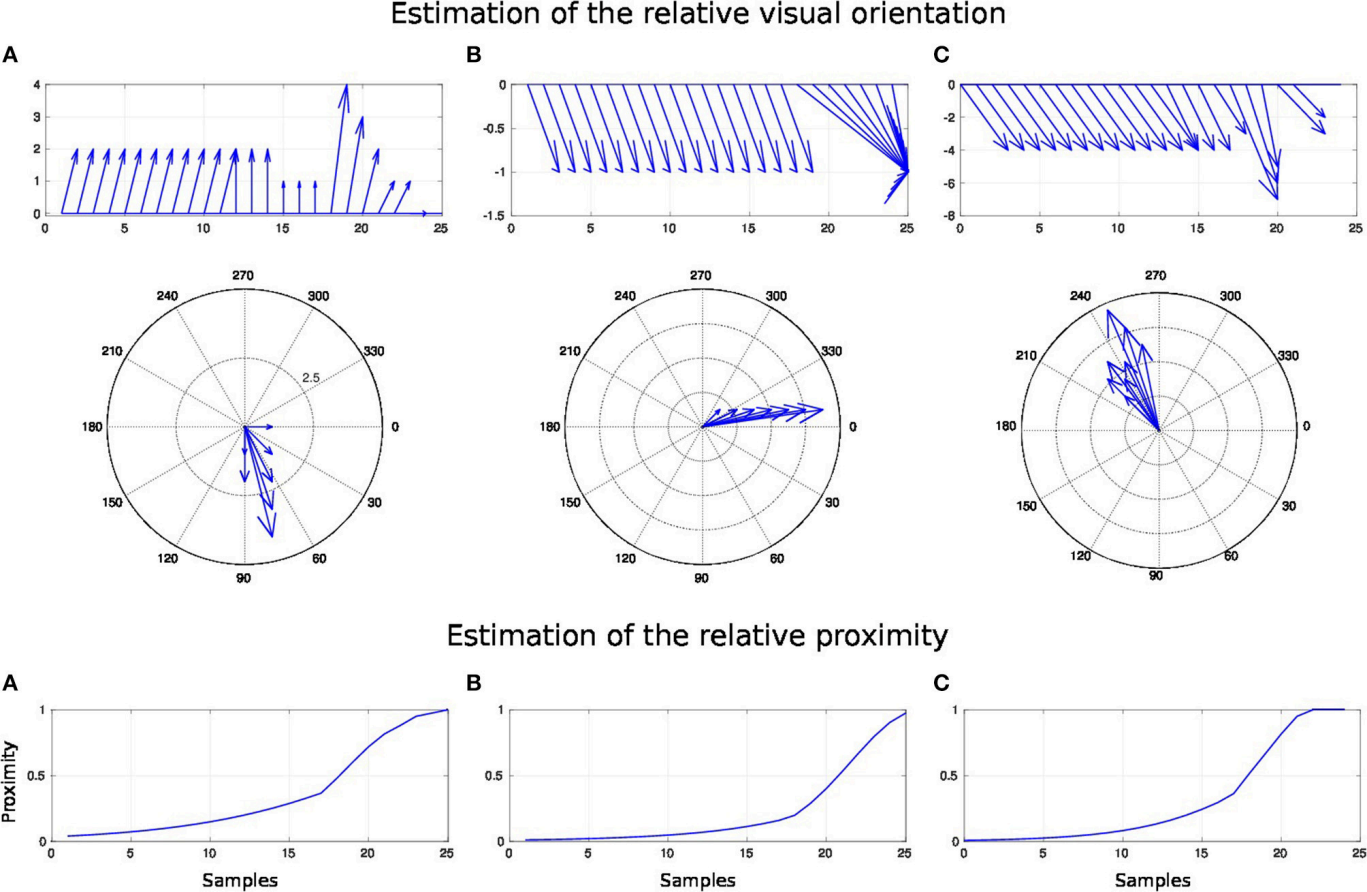
Spatial receptive fields and relative visual orientation of arm-centered GF neurons for three approaching targets. The bottom charts correspond to the estimated proximity of three arm-centered GF neurons respectively at locations **(A–C)**. The y axis corresponds to the amplitude level of the neurons and the x axis correspond to time iteration. The higher the amplitude level of the GF neurons is, The closer the target is with respect to the arm location. Each GF neuron has a different types of receptive field with respect to the arm part and the receptive fields are different from the target-centered GF neurons displayed in Figure 11. The middle and top charts represent the estimated relative visual orientation between the target and the robot arm. The density distribution of the estimated visual orientation is affined during displacement of the target toward the arm.

For this experiment, we fix the arm with the motor angle at 30°. Figure 12 shows the three starting points of the targets to the robotic arm. The paths are within the peripersonal space area and do not exceed it and each trajectory ends with contact with the skin. We plot in Figure 13 the estimated relative visual orientation in radians over time and in log-polar coordinates respectively in the top and middle charts as well as the estimated relative proximity to the arm in the bottom chart.

For the three paths, the relative visual orientation does not change when the target is distant from the arm, which corresponds to a low activity of the neurons (between 0.01 and 0.4). But putting the targets closer to the arm induces a more precise estimation of their orientation. Thus, in accordance with section 3.2, the orientation calculation gains in precision with respect to the distance to the arm. This is also true for the estimation of the targets' direction: as seen in the middle charts, the big arrows–, which correspond to the closest targets' positions,–indicate the optimal direction of the targets to reach the arm.

**Figure 14:**
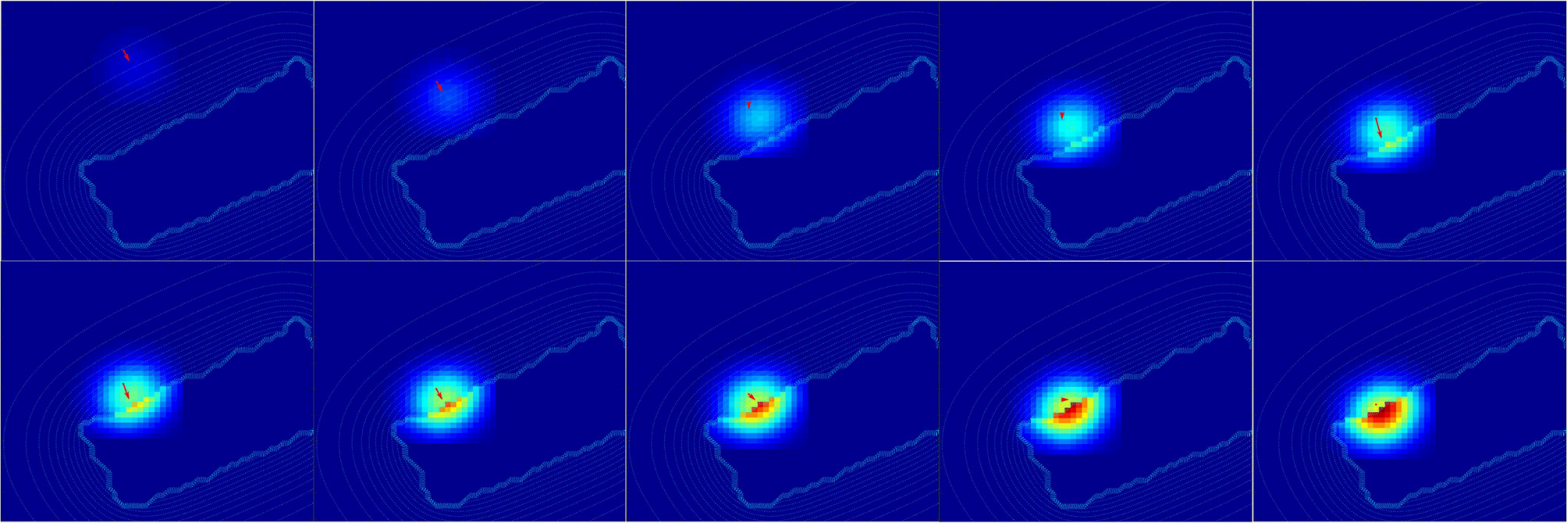
Spatial receptive fields and relative visual orientation of arm-centered GF neurons. Activity of arm-centered GF neurons relative to the target location for trajectory A.

For a better understanding, we present in Figure 14 the changes of spatial receptive fields and corresponding relative visual orientation of arm-centered GF neurons in detail for trajectory A. The arm-centered RF is calculated by the multiplication of the arm prediction in the eye-centered RF and target location in the eye-centered RF and relative visual orientation is taken from *argmax* function; see Part 3 in section 2.2.4.1. In the beginning, the orientation almost does not change when the receptive field is homogeneous, as seen in the first four subplots. But when the object is close to the arm, the orientation changes in correspondence with the more active neurons.

Once more, the analysis of the obtained results shows that the representation of the target location with respect to the arm is dynamic and constructed during the approaching of the target toward the arm.

## 4. Discussion

In this paper, we have proposed a brain-inspired model of multimodal neurons in the parietal cortex for the body representation of the robot arm Jaco and its peri-personal space. The neural model makes it possible to encode the location of the arm, the target and the relative distance between them in three different reference frames. This model is based on the integration of different modalities such as touch, vision and proprioception using the neural mechanism known as gain-modulation, which performs multiplicative interaction between variables. Such framework permits the dynamic coding of the body posture and targets in multiple coordinate systems even when the two systems are moving.This mechanism is particularly important for spatial interaction with objects and for solving spatial tasks online; e.g., tool-use, manipulation, dynamic coordination, interacting with someone else.

Before any target enters the peripersonal space of the robot, the arm and the target are coded in separate receptive fields: a receptive field centered on the artificial skin and another centered on the target in the visual space. As soon as the target enters the peripersonal space, the interaction between the two neural fields is computing a resulting receptive field (mutually referential), which makes it possible to estimate the relative distance and the relative visual orientation between the arm and the target. This behavior is similar to the one found in the parietal neurons and recorded by Bremner and Andersen (2012) and Bremner and Andersen (2014) for reaching tasks and by Iriki et al. (2001); Graziano and Botvinick (2002) for body image.

For instance, as soon as the robot moves toward or away the target, the spatial receptive fields of the neurons change and therefore the way targets are represented: in eye centered coordinates, in hand centered coordinates or in target centered coordinates. Thanks to the multiplication between the neural fields, the spatial resolution anchored at the arm becomes proportional to the vicinity of the target. Such computation may ease motor control and help also to create a sense of spatial awareness around the body, which is useful for constructing a notion of agency (Pitti et al., 2009a,b), of self and of intersubjectivity (Murata et al., 2016; Pitti, 2017).

As the GF mechanism serves the encoding of dynamical events, its lability due to multiplicative interaction across heterogeneous events may be advantageous for the construction of a plastic infant's body image during development (Gliga and Dehaene-Lambertz, 2005; Marshall and Meltzoff, 2015; Bhatt et al., 2016) as well as for the purpose of other cognitive tasks such as tool-use and body extension (Iriki et al., 1996; Murata et al., 2016), perspective-taking to have person-centered viewpoints (Iriki et al., 2001; Murata et al., 2016) or during perceptual illusions such as the rubber hand illusion, to mismatch visuo-tactile events in a confused body-centered representation (Botvinick and Cohen, 1998; Tsakiris et al., 2007).

In our previous research, we have modeled the visuo-tactile integration with neural networks using our artificial skin in order to study the rubber hand illusion although we did not have motor information at this time (Pitti et al., 2017). We think it is theoretically possible to simulate it as we will have a fast readaptation of the new motor position for the seen visual position of the fake hand as during the first phase of visuo-tactile based learning in our experiment. The learning between visual and proprioceptive information will be fast because it will be actively modulated by tactile stimulation as we proposed in Figure 4.

About the integration of an external tool to the body image, see Iriki et al. (1996). We think the adaptation mechanism may be similar also to the first phase of the visuo-tactile based learning of our experiments. If we connect a tool to any tactile position on the artificial skin–, a normal location would be on the robot hand if it has tactile sensors,– and a target touches the tool, a visuo-tactile integration will be done not on the skin surface but where the target is (at the tool location). We suggest that some 'tool' neurons may modify rapidly the third circuit in Figure 3 to model the “target-in-tool” centered reference frame, when we have the tool in hand, or even better, other maps may be created similar to this third circuit, each one specialized to a particular tool (Braud et al., 2018).

We think that our results are in line with observations showing how the peripersonal space increases when the subject is in motion (Noel et al., 2015; Bufacchi and Iannetti, 2018). Because gain-field neurons encode relative spatial information, they are effective either when objects are moving or when the body moves. In consequence, such mechanism may describe well spatial position of objects surrounding the body in motion. Since this construction is dynamic and depends on the context, peripersonal space remapping can work to certain limits only and spatial estimation may change also according to it. For instance, while the body moves, speed integration might be difficult for stabilization of incoming signals. Our framework may explain well how the remapping can be done of the third circuit in Figure 3 (as explained earlier for tool-use) to enlarge peripersonal space to the new context.

A different prediction can be made on phantom limbs with the observation that many amputees are feeling their phantom limb moving (Ramachandran and Blakeslee, 1998). If we think that tactile, visual and proprioceptive information are missing but the circuits for spatial representations are still there as after the first phase of visuo-tactile based learning, we may simulate the position of the arm moving in different coordinate systems (HE or TH).

Moreover, we suggest that the Gain-Field mechanism strongly supports the body schema construction during development.

Many research suggest that body knowledge occurs early in life and that the different modalities conspire to represent the body structure and nearby targets. Hock et al. found that infants as young as 3 months old are sensitive to the overall organization of body parts; (see Zieber et al., 2015; Hock et al., 2016; Jubran et al., 2018). Meltzoff et al. (2018) reports that the contralateral hand areas of the somatosensory cortex in 7-month-olds' is active during contact with the hands, suggesting neural structures represent hands early in life. Bremner et al. (2008) showed how 9-month-olds' use different strategies to perform reaching and grasping tasks by choosing the most effective modality (vision or touch) and RF.

Although we entrust strongly vision for representing space, the tactile information greatly enhances the calibration of a multi-centered referential system by connecting the visual and the proprioceptive information. This aspect is often neglected in neural models of reaching and motor control such as the ones proposed recently in Ajemian et al. (2001), Chang et al. (2009), Brayanov et al. (2012), Blohm (2012), and Ustun (2016) or those fewly emphasized as in Andersen (1997) and Baraduc et al. (2001).

The same is true in robotics and it is only recently that tactile information is taken into consideration. For instance, a color code (or a QR code) is often used to disambiguate between the target and the robot arm and to compute the relative distance between them. Robot architectures taking account of tactile information allow on the contrary to have a visual marker on the target only and to reconstruct back the visual position of the arm from tactile information. In our model, the position of the arm in the visual field is calculated via visual neurons (Perceptron units) that conditionally fire for conjunctive tactile and motor pattern. By doing so, they integrate tactile, visual, and proprioceptive information so that after the learning phase, these visual units are able to predict the visual location of the arm even without tactile input, just from the motor angle, where the arm has been touched by the target. This result is particularly interesting for motor simulation to anticipate contacts and to estimate the arm location even if the arm is occluded and in the dark.

For instance, one conclusion drawn by the Darpa Robotic Challenge is that all teams in the challenge failed to use aspects of the physical space to help their robots move (Atkeson et al., 2015). More contacts make tasks mechanically easier, but algorithmically more complicated. One full body artificial skin, however, is expected to be extremely useful as part of an early warning system to avoid errors and external disturbances.

Another use of tactile information is to ease motion control: as multiplicative neurons dynamically encode the location of objects relative to the robotic arm, the control task may be facilitated. The tactile sense may serve robots to perceive depth and calibrate the representation of the physical space relative to visual and motor modalities.

In our experiments, the camera was fixed and only the arm was moving. We think however that we can integrate this feature in the future. We did so partly in an earlier work based on audio-visual integration for eye-to-head change of reference frame with the head moving (Pitti et al., 2012). We think we can embed this feature using a similar network as the ones proposed in Andersen et al. (1985) and Salinas and Thier (2000) for visual and proprioceptive integration using GF neurons. The vestibular information can be useful as well and in line with evidences from neuroscience.

Because gain-field neurons encode a relative spatial information, they are effective either when objects are moving or when the body moves. In consequence, our architecture may describe well spatial position of objects with the body in motion.

Although our experiences are currently performed in 2D space and has been applied with one single degree of freedom only, and without taking account of the object shape (its affordance), we do not see any constraints to extend this framework to 3D reach and grasp. As it is known that the orientation of the hand, depth perception and the object shape are required for 3D grasping, many results emphasize the role of gain modulation also for it. For instance, Kakei and colleagues found that the control of the forearm muscles for pronation/suppination are coded with parieto-motor neurons sensitive to visual directions (Kakei et al., 2003) as it is for arm motion. Experiments studying the hand orientation with oriented grippers showed also the importance of gain modulation for dynamically aligning the hand to the target orientation in the vertical plan (Baumann et al., 2009; Fluet et al., 2010) or for reaching objects aligned in various 3D orientations (Sakata et al., 1997; Murata et al., 2016). Furthermore, “depth neurons” have been found in the parietal cortex for the visual control on hand action (Rizzolatti et al., 1997; Sakata et al., 1997; Filippini et al., 2018). Sakata et al. (1997) suggested that depth movement is encoded from the associative interaction between size change and disparity change in the visual field and (Ferraina et al., 2009) proposed further that the GF mechanism supports the integration of hand movement depth for encoding of hand position and movement in 3D space.

Some recent robotic results found that it is possible to reconstruct back the 3D information of objects (Eslami et al., 2018) or to estimate their physics through observation, without interactions and from huge visual data only (Yildirim et al., 2017). Despite these impressive results, we believe nonetheless that embodiment –that is, the sensorimotor information structure of agents,– is mostly missing in these works in order for one agent to construct a unified and amodal spatial representation of the body. In future works, we will attempt to extend our framework to 3D space, toward learning the affordance of objects and interacting with them.

## Author Contributions

GP designed and made the experiments and wrote the paper. AP designed experiments and wrote the paper. OT and PG supervised the research.

### Conflict of Interest Statement

The authors declare that the research was conducted in the absence of any commercial or financial relationships that could be construed as a potential conflict of interest.

## Supplemental Data

Some complementary videos of Experiment 1 and 2 are provided at location: http://perso-etis.ensea.fr/alexpitt/PPS_files.
